# Late-Onset Autoimmune Myasthenia Gravis: A Diagnosis Not to Be Overlooked in the Elderly

**DOI:** 10.7759/cureus.82707

**Published:** 2025-04-21

**Authors:** Abrar-Ahmad Zulfiqar

**Affiliations:** 1 Geriatrics, University Hospital of Strasbourg, Strasbourg, FRA

**Keywords:** anti-achr antibodies, autoimmune myasthenia, bulbar involvement, elderly, ptosis

## Abstract

Autoimmune myasthenia gravis is a rare neuromuscular disorder of autoimmune origin, characterized by fluctuating muscle weakness. Although it classically occurs in young adults, late onset in the elderly is possible, often with atypical presentations that may delay diagnosis. We report the case of an 81-year-old female patient who initially consulted us with fluctuating right ptosis, in the absence of other neurological signs. Biological workup revealed the presence of anti-acetylcholine receptor (anti-AChR) antibodies, confirming the diagnosis of autoimmune myasthenia gravis. Thoracic imaging revealed no thymoma. Anticholinesterase therapy was initiated with partial improvement, followed by clinical progression two years later to bulbar involvement (dysarthria, swallowing disorders). Management involved the administration of intravenous immunoglobulins, with a favorable clinical response. Corticosteroid therapy was then introduced, together with azathioprine immunosuppressive therapy, stabilizing the course. This case illustrates the importance of considering the diagnosis of myasthenia gravis, even at an advanced age, in the face of fluctuating or unusual signs. It also highlights the diagnostic and therapeutic challenges specific to the geriatric population, particularly in relation to comorbidities, adverse drug reactions, and the need for multidisciplinary follow-up. Early recognition and treatment of the disease can significantly improve prognosis and quality of life.

## Introduction

Autoimmune myasthenia gravis (MG) is a rare neuromuscular disorder of autoimmune origin, characterized by fluctuating muscle weakness, often aggravated by exercise and improved by rest. While it typically affects young and middle-aged adults, it can also present late in life in elderly subjects, although it is less frequent and sometimes confusing in its clinical presentation. MG is an autoimmune disorder characterized by the production of autoantibodies that target components of the neuromuscular junction, most commonly the acetylcholine receptor (AChR), leading to impaired synaptic transmission and fluctuating muscle weakness. In late-onset cases, typically defined as onset after the age of 50, the disease may present diagnostic and therapeutic challenges due to overlapping symptoms with other age-related conditions. The diagnosis in older adults can be delayed or mistaken for other age-related conditions, such as stroke, motor neuron disease, or even functional decline. In this population, atypical symptoms such as isolated dysphagia, dysphonia, or general fatigue may predominate, contributing to underdiagnosis or misdiagnosis [1]. Moreover, elderly patients may present with multiple comorbidities and polypharmacy, further complicating the clinical picture and therapeutic management [2].

The study by Tang et al. examined 1,160 Chinese patients with MG, categorizing them into early-onset (18-49 years), late-onset (50-64 years), and very-late-onset (≥65 years) groups [3]. Findings indicated that very-late-onset MG patients were predominantly male, often presented with ocular MG, and had higher rates of AChR and titin antibody positivity. This group exhibited poorer prognoses, with fewer achieving minimal manifestation status, shorter durations of symptom control, and higher MG-related mortality compared to younger cohorts. Notably, the absence of immunotherapy was associated with worse outcomes in very-late-onset patients, underscoring the importance of tailored treatment strategies for the elderly [4]. 

The prevalence of late-onset MG appears to be increasing, possibly due to population aging and improved awareness. It is also associated more frequently with anti-AChR antibodies and tends to follow a different course compared to early-onset forms. Management in the elderly requires balancing immunosuppressive treatment efficacy with the risks of adverse effects, which are often more pronounced in this population. Therefore, a high index of suspicion is necessary when encountering unexplained neuromuscular symptoms in older adults. Here's a case study to illustrate the problem.

## Case presentation

This is an 81-year-old patient with a notable history of osteoporosis fracture (femoral T-score -3.3 and spinal T-score -3.0, treated with Aclasta between 2016 and 2018), a femoral neck fracture in 2015, and an old uterine leiomyoma. She consulted in March 2018 for fluctuating right ptosis evolving for 15 days, accompanied by unusual headaches. The clinical examination was strictly normal, including neurovascular findings. In fact, on initial examination, the patient presented with right ptosis but no dysphonia, without limb weakness or respiratory involvement. Deep tendon reflexes were preserved, and there were no sensory deficits or signs of central nervous system involvement. The absence of diurnal variation in symptoms or cerebellar signs helped rule out stroke, Parkinsonism, or other neurodegenerative causes.

Given the isolated but atypical ophthalmic symptomatology, a workup was initiated. Doppler examination of the supra-aortic trunks showed no carotid dissection or significant stenosis. An autoimmune workup was ordered to rule out autoimmune MG: presence of anti-AChR antibodies at 7.20 nmol/L (positive >0.40), in the absence of anti-MuSK antibodies and the absence of the anti-titin antibody. Anti-smooth muscle antibodies were negative, although antinuclear antibodies were positive.

A thoracic CT scan was performed to rule out a thymoma. The examination, although initially without iodine injection, was completed by an injected acquisition and showed no thymic mass or mediastinal adenopathy. A referral to a neurologist concluded that there was no indication for corticosteroid therapy, given the isolated ophthalmic involvement. An anticholinesterase treatment was started: ambenonium (Mytélase®), 10 mg three times a day.

In the absence of improvement, treatment was changed after one month to pyridostigmine (Mestinon® 60 mg) morning and evening, resulting in a clear improvement in ptosis, with only discomfort in the upper right gaze.

Two years later, the patient presented with gradually worsening fluctuating dysarthria, associated with incipient swallowing difficulties. The AChR antibody level was significantly increased to 17.60 nmol/L. A neurological opinion led to an increase in the dose of Mestinon, which caused iatrogenic bladder hyperactivity and urinary incontinence. A urethrovesical fibroscopy was carried out with no particular findings, and treatment doses were adjusted to one tablet three times a day.

One year later, the patient presented with a myasthenic crisis characterized by hypophonia, swallowing disorders, and dysarthria, requiring hospitalization. Examination revealed a bladder globe treated by indwelling catheterization. Cerebrovascular imaging ruled out ischemic stroke and carotid dissection. AChR antibody level was 16.40 nmol/L. An immunoglobulin infusion (Privigen® 40 g) was administered with an excellent clinical response: disappearance of bulbar signs and recovery of autonomy.

A new neurological opinion confirmed seropositive MG with bulbar involvement, having responded well to immunoglobulins. Oral corticosteroid therapy was started at increasing doses up to 35 mg/day, with gradual tapering off once improvement was achieved, followed by azathioprine, titrated to 2 mg/kg/day. Repetitive nerve stimulation showed a decremental response consistent with MG. Clinical response was monitored using the Quantitative Myasthenia Gravis (QMG) score, which improved from 11 to 4 over three months. Since then, clinical evolution has been stable.

The timeline below summarizes the main medical events in this clinical case of late-onset autoimmune MG in an elderly patient. It provides a clear visual overview of symptom progression, diagnostic assessments, and therapeutic interventions over time (Table 1).

**Table 1:** Clinical timeline of autoimmune myasthenia gravis anti-AChR: anti-acetylcholine receptor; IVIg: intravenous immunoglobulin

2018	2020	2021	2022
Fluctuating right ptosis	Bulbar symptoms begin	Myasthenic crisis	Long-term treatment
			Corticosteroids → azathioprine
Anti-AChR + (7.20 nmol/L)	Dysarthria, swallowing issues	Hypophonia, dysphagia	
			Clinical stabilization
Mytélase → Mestinon	AChR ↑ 17.60 nmol/L	Hospitalization	
No thymoma	Mestinon adjusted	IVIg (Privigen®) → recovery	

## Discussion

Autoimmune MG is a rare acquired pathology, with an estimated incidence of 1-2 cases per 100,000 inhabitants per year. It can occur at any age, but its presentation in the elderly is often misleading and may delay diagnosis [5].

In this case, the 81-year-old patient presented with an initial ophthalmic form with isolated ptosis, which is a frequent presentation of MG, particularly in the elderly [6]. The purely ophthalmic form is found in around 15% of cases at diagnosis, but may remain restricted to this territory in a third of patients [7].

The diagnosis was confirmed by the presence of anti-AChR antibodies, found in around 85% of generalized forms and 50% of purely ophthalmic forms [8]. In this case, positive serology with a significant level at 7.20 nmol/L warranted symptomatic treatment with anticholinesterase. Secondary transition to a bulbar form is a classic evolution, particularly in elderly patients [9].

The absence of thymoma on thoracic CT is an important factor. Indeed, while a thymoma is found in 10-15% of cases, it is mostly present in earlier-onset forms. Late-onset forms are more often associated with involutional or atrophic thymia with no visible mass [10].

Anticholinesterase therapy was initially only partially effective, but had to be adapted due to side effects (bladder hyperactivity). Progression to a myasthenic crisis with dysphonia, dysarthria, and swallowing disorders is a serious feature requiring emergency treatment. The rapid response to intravenous immunoglobulin (IVIg) administration is in line with current recommendations, which place IVIg or plasma exchange as first-line treatment in severe or decompensated forms [11].

Finally, the secondary introduction of background corticosteroid therapy followed by immunosuppressive therapy (azathioprine) is one of the recommended therapeutic strategies for generalized or recurrent bulbar forms. Azathioprine is a first-line immunosuppressant in refractory or cortico-dependent MG, with a delay of action of several months but good tolerance in elderly subjects [7,10]. Sahai et al. conducted a retrospective study of seven patients with AChR antibody-positive MG, all treated with rituximab. This work highlights the safety and efficacy of rituximab in late-onset MG. However, more studies are needed to clarify the use of rituximab in the elderly [11].

## Conclusions

This case illustrates the importance of prolonged follow-up, individualized therapeutic adaptation, and diagnostic vigilance in the face of apparently benign symptoms in the elderly. MG may initially be ophthalmic, evolve into a bulbar form, and respond favorably to appropriate therapies even after a late diagnosis. This case highlights the importance of considering MG in elderly patients presenting with isolated bulbar or ocular symptoms. Early electrophysiological testing and antibody screening are essential for prompt diagnosis. Clinicians should maintain a high index of suspicion and initiate immunotherapy without delay to improve outcomes. 
